# Multimodal Large Language Model for Fracture Detection in Emergency Orthopedic Trauma: A Diagnostic Accuracy Study

**DOI:** 10.3390/diagnostics16030476

**Published:** 2026-02-03

**Authors:** Sadık Emre Erginoğlu, Nuri Koray Ülgen, Nihat Yiğit, Ali Said Nazlıgül, Mehmet Orçun Akkurt

**Affiliations:** Department of Orthopedics and Traumatology, Ankara Sincan Training and Research Hospital, Sincan, Ankara 06935, Türkiye

**Keywords:** emergency department, orthopedic trauma, fracture detection, large language model, artificial intelligence, radiography

## Abstract

**Background**: Rapid and accurate fracture detection is critical in emergency departments (EDs), where high patient volume and time pressure increase the risk of diagnostic error, particularly in radiographic interpretation. Multimodal large language models (LLMs) with image-recognition capability have recently emerged as general-purpose tools for clinical decision support, but their diagnostic performance within routine emergency department imaging workflows in orthopedic trauma remains unclear. **Methods**: In this retrospective diagnostic accuracy study, we included 1136 consecutive patients referred from the ED to orthopedics between 1 January and 1 June 2025 at a single tertiary center. Given the single-center, retrospective design, the findings should be interpreted as hypothesis-generating and may not be fully generalizable to other institutions. Emergency radiographs and clinical data were processed by a multimodal LLM (2025 version) via an official API using a standardized, deterministic prompt. The model’s outputs (“Fracture present”, “No fracture”, or “Uncertain”) were compared with final diagnoses established by blinded orthopedic specialists, which served as the reference standard. Diagnostic agreement was analyzed using Cohen’s kappa (κ), sensitivity, specificity, accuracy, and 95% confidence intervals (CIs). False-negative (FN) cases were defined as instances where the LLM reported “no acute fracture” but the specialist identified a fracture. The evaluated system is a general-purpose multimodal LLM and was not trained specifically on orthopedic radiographs. **Results**: Overall, the LLM showed good diagnostic agreement with orthopedic specialists, with concordant results in 808 of 1136 patients (71.1%; κ = 0.634; 95% CI: 68.4–73.7). The model achieved balanced performance with sensitivity of 76.9% and specificity of 66.8%. The highest agreement was observed in knee trauma (91.7%), followed by wrist (78.8%) and hand (69.6%). False-negative cases accounted for 184 patients (16.2% of the total cohort), representing 32.4% of all LLM-negative assessments. Most FN fractures were non-displaced (82.6%), and 17.4% of FN cases required surgical treatment. Ankle and foot regions showed the highest FN rates (30.4% and 17.4%, respectively), reflecting the anatomical and radiographic complexity of these areas. Positive predictive value (PPV) and negative predictive value (NPV) were 69.4% and 74.5%, respectively, with likelihood ratios indicating moderate shifts in post-test probability. **Conclusions**: In an emergency department-to-orthopedics consultation cohort reflecting routine clinical workflow, a multimodal LLM demonstrated moderate-to-good diagnostic agreement with orthopedic specialists, broadly within the range reported in prior fracture-detection AI studies; however, these comparisons are indirect because model architectures, training strategies, datasets, and endpoints differ across studies. However, its limited ability to detect non-displaced fractures—especially in anatomically complex regions like the ankle and foot—carries direct patient safety implications and confirms that specialist review remains indispensable. At present, such models may be explored as hypothesis-generating triage or decision-support tools, with mandatory specialist confirmation, rather than as standalone diagnostic systems. Prospective, multi-center studies using high-resolution imaging and anatomically optimized algorithms are needed before routine clinical adoption in emergency care.

## 1. Background

Orthopedic traumas constitute a significant proportion of emergency department visits, and rapid, accurate diagnosis in these patients is a critical component of treatment success [1,2]. In the emergency department setting, high patient volume and time constraints can increase the risk of error, particularly in radiological evaluation processes [3]. Therefore, there is a need for supportive systems that enable the rapid and reliable interpretation of imaging data.

In recent years, artificial intelligence (AI)-based algorithms have made remarkable progress in the field of medical imaging, particularly with the advancement of deep learning techniques [4,5]. In radiology practice, the use of AI applications in areas such as fracture detection, evaluation of joint pathologies, and automatic classification of musculoskeletal images is becoming increasingly widespread [6,7]. AI-assisted systems have been reported to achieve high accuracy rates in various diagnostic domains within musculoskeletal imaging, including both fracture detection and bone density assessment [8,9]. However, diagnostic performance can be influenced by factors such as different anatomical regions, image quality, lesion type, and clinical scenarios [10,11]. While most existing AI tools for fracture detection are trained as task-specific, narrow models, recently emerged multimodal large language models (LLMs) offer a general-purpose approach capable of processing both images and clinical text. To date, no large-scale study has evaluated the real-time diagnostic performance of such a multimodal LLM in emergency orthopedic radiography. Specifically, prior fracture-detection AI literature largely focuses on task-specific models, whereas evidence remains limited on how a general-purpose multimodal LLM—without dedicated fracture-training—performs when applied to real emergency radiographs and brief clinical text. Unlike task-specific convolutional neural networks (CNNs) and vision transformers that are trained and optimized exclusively for fracture detection, vision-capable multimodal large language models (LLMs) are general-purpose systems designed to integrate images with free-form clinical text across heterogeneous tasks. This generality may offer practical advantages (e.g., flexible multimodal interaction and easier adaptation to diverse clinical questions) but is expected to come at the cost of lower task-specific optimization compared with dedicated fracture-detection models. Therefore, evaluating a general-purpose multimodal LLM in emergency orthopedic radiography is clinically relevant to define its realistic strengths and limitations as decision support, rather than to position it as a replacement for specialist interpretation or for state-of-the-art task-specific fracture-detection systems.

The potential of AI in emergency orthopedic practice is significant, both for accelerating the diagnostic process and reducing physician workload. However, the integration of AI systems into clinical practice requires evaluation of their performance on real patient data, determination of their reliability, and identification of potential limitations [12,13].

This study investigated the diagnostic agreement of a general-purpose multimodal LLM in patients referred from the emergency department to orthopedics; results were evaluated according to anatomical region and time of presentation. Furthermore, by analyzing the clinical characteristics of cases where the AI failed to diagnose, inferences were made regarding the use of this technology in emergency orthopedic practice. Our study is one of the first large-scale, clinically data-driven investigations to evaluate the diagnostic performance of a general-purpose, multimodal large language model (LLM), as opposed to a narrowly trained AI, for fracture detection in emergency orthopedic evaluation.

## 2. Methods

### 2.1. Ethics Approval and Patient Selection

This retrospective study was approved by the Ankara Sincan Training and Research Hospital Clinical Research Ethics Committee (Protocol No: SEAH-BAEK-2025-103).

A total of 1263 consecutive patients consulted from the emergency department to the orthopedic clinic between 1 January and 1 June 2025 were screened. Both adult and pediatric patients were eligible for inclusion; patient age was recorded as a baseline characteristic. A total of 127 patients were excluded from the analysis based on the following criteria:

- Multiple trauma: *n* = 45
- Presence of orthopedic implants: *n* = 38
- Inadequate image quality: *n* = 29
- Missing clinical data: *n* = 15

Examinations with incomplete standard radiographic projections (e.g., missing anteroposterior or lateral views) were excluded during screening. Inadequate image quality was defined as radiographs with marked motion blur, insufficient exposure or contrast limiting cortical margin assessment, severe positioning/rotation artifacts, or other technical limitations that precluded confident evaluation of the suspected anatomical region.

The cohort reflects routine emergency department-to-orthopedics consultation workflow at our institution, using consecutively referred cases over the study period. As this was a retrospective analysis, the study did not assess workflow impact outcomes such as time-to-diagnosis or changes in clinical decision-making.

**Figure 1:**
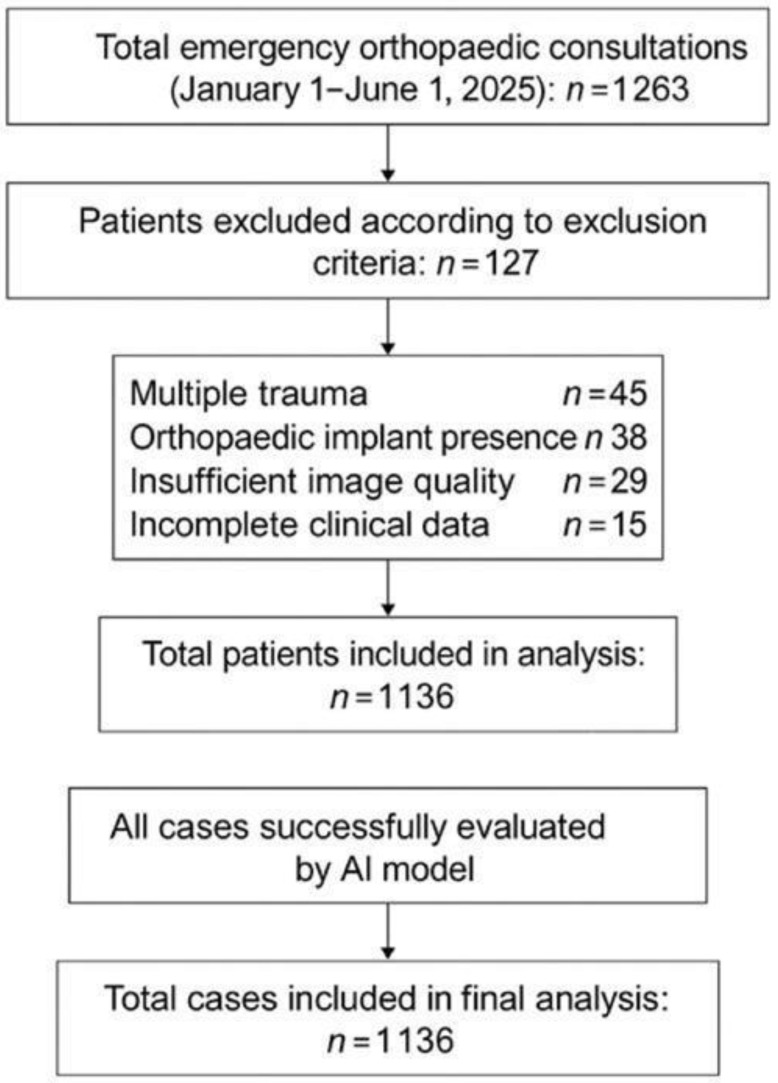
Flowchart illustrating the patient selection process.

Consequently, 1136 patients were included in the study. Due to the retrospective design, individual patient consent was not obtained; all data were anonymized before analysis. The patient selection process is summarized in the study flowchart (Figure 1).

### 2.2. Artificial Intelligence Assessment Protocol

All radiographs were evaluated using a multimodal large language model with image-recognition capability (ChatGPT-5, OpenAI, multimodal vision-enabled model, 2025) via the official API. Assessments were performed via the model’s official API using a standardized prompt template, with the temperature parameter set to 0 to minimize randomness and ensure deterministic, reproducible outputs.

Radiographs were retrieved from the hospital Picture Archiving and Communication System (PACS) and exported for research purposes after anonymization. All images were anonymized prior to processing and transmitted to the model via a secure API for inference only. No identifiable patient information was included in the API requests. The multimodal LLM received the radiographic image together with a standardized text prompt in which the placeholder [anatomical region] was replaced by the specific anatomical region (including laterality when applicable; e.g., ‘right wrist’). No additional clinical or demographic information (e.g., mechanism of injury, symptoms, age/sex) was provided. The model then generated a text-only categorical output. All radiographs were submitted as independent, case-level API requests using a fixed prompt and deterministic settings (temperature = 0), without session carryover, conversational context, or batch-level processing; each image was therefore evaluated in isolation, and ordering effects are not expected to influence outputs. No visual overlays, markings, or heatmaps were produced by the model. All AI assessments were performed retrospectively and were not used for real-time clinical decision-making or shared with patients. The evaluated LLM was used exclusively for research in a retrospective analysis and is not presented as an FDA- or CE-approved fracture-detection device for clinical deployment.

To minimize the effect of radiographic acquisition variability, we applied predefined technical inclusion criteria. Only examinations containing at least two standard projections (anteroposterior and lateral) with adequate image resolution were included. Studies with missing projections, non-standard views, or insufficient image quality were excluded during screening. For examinations with two standard projections (anteroposterior and lateral), images were evaluated sequentially for the same anatomical region rather than being provided as a combined multi-view input in a single request; each projection was assessed independently using the same prompt structure. Therefore, radiographic quality was largely standardized across the cohort and was not treated as an independent variable in the analysis. Radiographs were exported from the PACS in their original diagnostic resolution. No additional compression, resizing, or image enhancement was applied prior to transmission via the API beyond standard platform requirements.

“Evaluate the following [anatomical region] radiograph for the presence of an acute fracture.

Provide your response ONLY as one of the following options: ‘Fracture present’, ‘No fracture’, ‘Uncertain’.”

The anatomical region (e.g., “knee”, “wrist”) was specified in the prompt for each image. The response format was standardized; free-text responses were not included in the study.

Anatomical regions were defined according to the primary radiographed body part associated with the clinical complaint. Laterality (left/right) was recorded for each examination. If multiple fractures were present within the same anatomical region, the case was counted once for that region. Cases involving injuries to more than one anatomical region were excluded under the multiple-trauma criterion.

Patient age, mechanism of injury, and additional clinical context were intentionally excluded from the prompt to isolate the model’s image-based fracture detection performance and to avoid introducing subjective clinical priors.

### 2.3. Clinical Evaluation (Reference Standard)

In our emergency department, formal radiology reports are not routinely available at the time of initial evaluation for acute orthopedic trauma. Therefore, the first-line interpretation of radiographs and clinical decision-making are performed by on-call orthopedic surgeons as part of standard care. For this study, the reference standard diagnosis was established independently by two orthopedic specialists (mean 12 years’ experience, range 6–21) who were blinded to the AI outputs. Inter-observer agreement was excellent (κ = 0.89). In cases of disagreement (*n* = 46, 4.1%), a third senior orthopedic specialist adjudicated, and a consensus diagnosis was reached. CT/MRI confirmation was not performed systematically; advanced imaging was obtained only in selected cases per clinical judgment, which may have influenced verification of subtle fractures.

### 2.4. Statistical Analysis

The null hypothesis of this study was that there is no agreement beyond chance between the fracture detection results of the multimodal LLM and specialist orthopedic physicians. Diagnostic agreement was primarily evaluated using Cohen’s kappa statistic to test this hypothesis. A kappa value significantly greater than zero was interpreted as evidence to reject the null hypothesis and indicate meaningful diagnostic agreement beyond chance.

No a priori power analysis was performed because this was a retrospective study based on a consecutive cohort over a fixed time period. The sample size therefore reflects the number of eligible examinations available after applying predefined exclusion criteria.

Diagnostic performance was assessed using the following metrics:

- Cohen’s kappa (κ)
- Sensitivity, Specificity
- Positive Predictive Value (PPV), Negative Predictive Value (NPV)
- Overall accuracy
- F1-score
- Likelihood ratios (+LR, −LR)

For the primary diagnostic performance analysis, “Uncertain” outputs were conservatively grouped with negative assessments (“No fracture”).

All diagnostic test metrics were reported with 95% confidence intervals (Wilson score method). Confidence intervals were calculated specifically for sensitivity, specificity, PPV, and NPV.

- Categorical variables: Chi-square test
- Continuous variables: Student’s *t*-test
- Statistical significance: *p* < 0.05

Analyses were performed using SPSS 28.0 (IBM Corp., Armonk, NY, USA) and Python 3.10 (scikit-learn, pandas libraries).

The reporting of this diagnostic accuracy study was informed by the STARD-AI recommendations, where applicable, to enhance transparency and reproducibility.

Region-based and time-of-presentation subgroup analyses were exploratory; no formal adjustment for multiple comparisons was applied, and findings should be interpreted descriptively.

## 3. Results

### 3.1. General Diagnostic Agreement

The preliminary diagnoses provided by the AI (LLM) were compared against the final diagnoses made by the orthopedic specialist in 1136 patients consulted from the emergency department. The overall diagnostic agreement between the LLM and orthopedic specialists is summarized in Table 1. Complete agreement was found in 808 cases (71.13%) (Cohen’s kappa = 0.634; 95% CI: 68.42–73.69).

False-positive cases were defined as examinations in which the AI system reported “fracture present” while the orthopedic specialist diagnosis was “no fracture.” Based on the model specificity, the corresponding false-positive rate was 33.2%. The overall diagnostic accuracy of the model across all anatomical regions was 71.1%, as summarized in Table 2.

Sensitivity analysis for indeterminate outputs: In our primary analysis, “Uncertain” outputs were conservatively grouped with “No fracture”. To assess the impact of this classification, we performed a sensitivity analysis in which all 16 “Uncertain” outputs were reclassified as “Fracture present” (among these, 8 were true fractures and 8 were non-fractures according to the reference standard). This reclassification increased sensitivity from 76.9% to 77.9%, decreased specificity from 66.8% to 64.3%, and reduced the false-negative rate (percentage of total cohort) from 16.2% to 15.5%. These changes illustrate the trade-off in performance metrics based on the handling of indeterminate model outputs.

The general diagnostic performance metrics of the model are summarized in Table 2. LLM demonstrated a good level of agreement with orthopedic specialists, with balanced sensitivity (76.9%) and specificity (66.8%) values. Region-based diagnostic agreement findings are presented in Table 3, and diagnostic agreement by time of presentation is summarized in Table 4. A minimum of 30 cases per anatomical region was used to reduce instability of region-level estimates and to avoid over-interpreting very small subgroups. Given the smaller sample sizes in some anatomical subgroups, region-level estimates should be interpreted cautiously; Table 3 reports 95% confidence intervals for agreement, and we additionally provide region-wise error composition (false-positive and false-negative counts) in Supplementary Table S2 to contextualize precision and safety-relevant failure patterns.

In the reference standard, the overall fracture prevalence was high in this ED-to-orthopedics consultation cohort (binary-set prevalence: 56.4%), consistent with an enriched population compared with unselected ED radiograph screening; therefore, PPV/NPV should be interpreted in this prevalence context (Supplementary Table S1).

### 3.2. Analysis of False-Negative (FN) Cases

**Figure 2:**
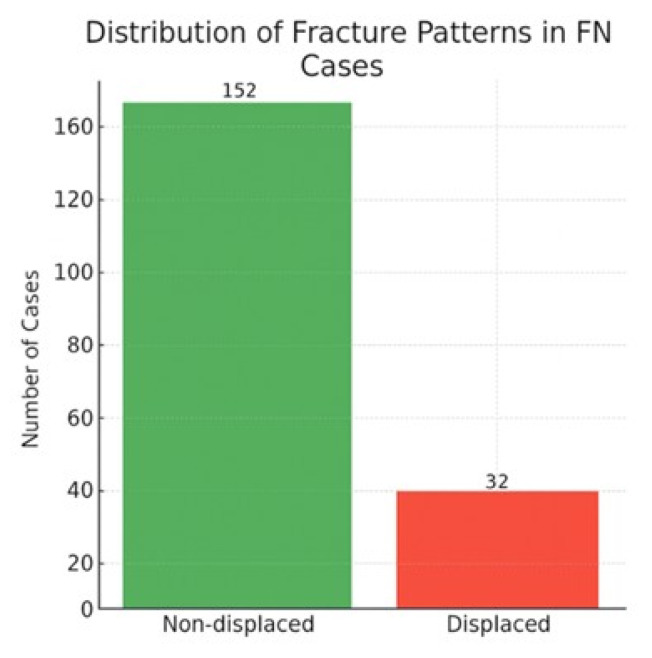
Distribution of fracture patterns in false-negative (FN) cases.

Cases where the AI assessed as ‘no acute, significant fracture detected’ but the specialist diagnosed ‘fracture present’ were considered FN. Out of the total 1136 cases, the AI reported no acute significant fracture in 568 cases (50.0%). Among these AI-negative cases, the specialist identified a fracture in 184 cases, representing a false-negative rate of 32.4% among the AI’s negative assessments (and 16.2% of the total cohort). A regional analysis of diagnostic discrepancies revealed that in ankle trauma, all instances of disagreement (56/56, 100%) were attributable to false-negative assessments by the AI, with no false-positive cases observed in this anatomical region. Among surgically treated false-negative cases, the most commonly missed fracture types were pediatric supracondylar fractures of the distal humerus and adult femoral neck fractures, indicating that a subset of missed cases involved clinically high-risk injuries requiring prompt operative management. The distribution and characteristics of false-negative cases are summarized in Table 5, and the fracture pattern distribution among false-negative cases is illustrated in Figure 2.

Diagnostic outcome distribution is provided in Supplementary Table S1, and region-wise error composition (false-positive and false-negative counts) is provided in Supplementary Table S2. All radiographs were fully de-identified, and any visual annotations are included solely for illustrative purposes. In most false-negative cases, fractures were retrospectively visible on plain radiographs but were subtle, non-displaced, or partially obscured by overlapping anatomical structures; a minority required advanced imaging for definitive confirmation.

**Figure 3:**
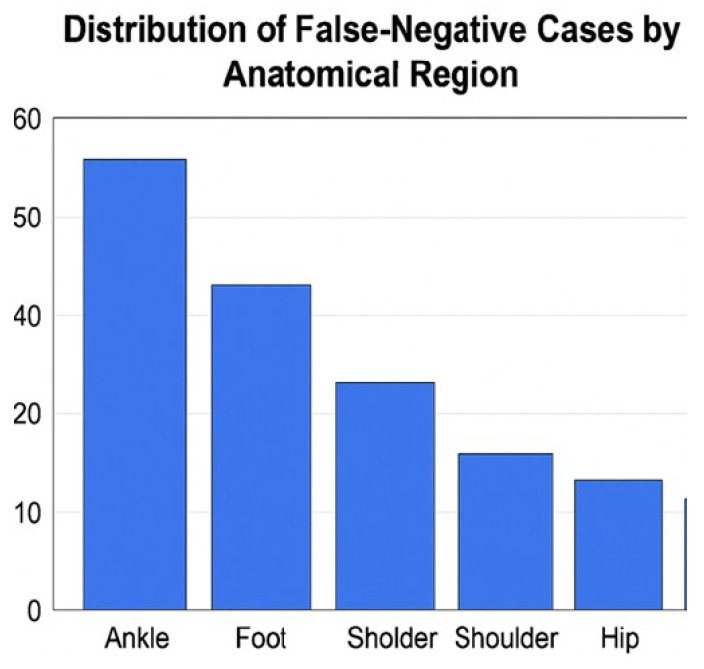
Regional distribution of false-negative (FN) cases according to anatomical region.

**Figure 4:**
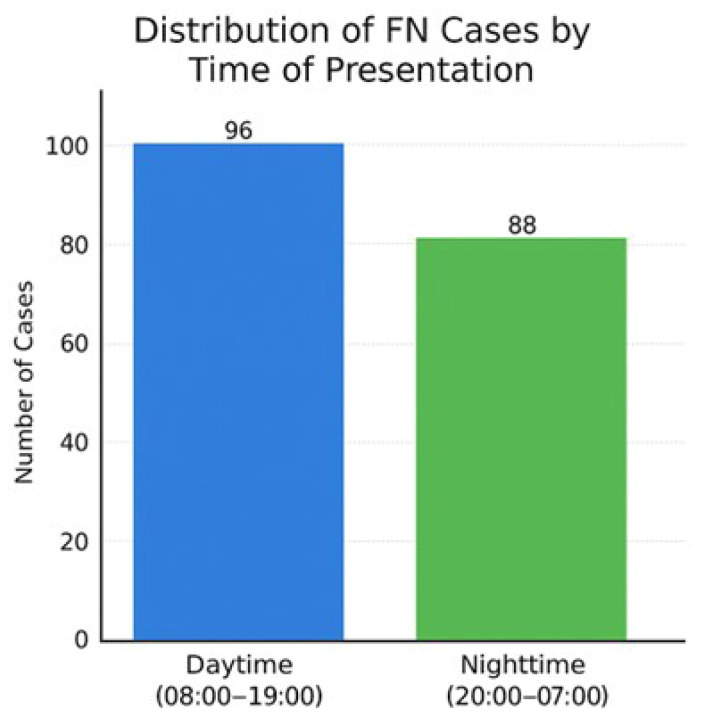
Distribution of false-negative (FN) cases by time of presentation.

In the regional and temporal analyses of false-negative (FN) cases, notable variability was observed across anatomical regions and consultation times. The highest FN rates occurred in the ankle (30.4%) and foot (17.4%) regions, likely reflecting the radiographic complexity of these areas due to overlapping small bones and low soft-tissue contrast that can obscure subtle, non-displaced fracture lines. In contrast, the distribution of FN cases by consultation time was relatively uniform between daytime (52.2%) and nighttime (47.8%) evaluations. This suggests that AI performance was not significantly affected by the time of examination but was strongly influenced by regional anatomy (Figure 3 and Figure 4).

## 4. Discussion

In this study, the diagnostic agreement of an AI-based system (LLM) was evaluated in 1136 patients consulted from the emergency department to orthopedics, and the overall agreement rate was found to be 71.13% (Cohen’s κ = 0.634). This rate is consistent with many AI-based orthopedic imaging studies reported in the literature. For example, Lindsey et al. [6] reported that a deep learning algorithm achieved 80.8% accuracy in detecting wrist fractures, while Olczak et al. [7] reported the overall accuracy of AI in orthopedic trauma radiographs as 73.7%. The kappa value of 0.63 in our study corresponds to a “good” level of agreement according to the Landis and Koch classification [14]. The likelihood ratios indicate that the model provides only moderate shifts in post-test probability, supporting use as decision support rather than a stand-alone rule-in or rule-out test. This is consistent with the observed false-negative burden, particularly for non-displaced fractures.

When evaluated by anatomical region, the highest agreement rate (91.67%) was obtained in knee trauma. The literature also reports that AI systems show high performance in evaluating knee joint radiographs [15]. This can be attributed to the fact that knee fractures typically present with distinct radiological findings and high image quality enhances diagnostic accuracy. Agreement in wrist and hand trauma was 78.79% and 69.57%, respectively, which is similar to the 70–82% range reported in previous studies [7,16]. However, the relatively lower agreement in more complex anatomical regions such as the shoulder and ankle indicates that AI needs improvement in these areas. The absence of false-positive cases in ankle trauma suggests a conservative decision threshold in this region, which may reduce overcalling at the expense of increased false-negative risk.

One of the important findings of our study is that non-displaced fractures constituted 82.61% of the AI’s false-negative (FN) cases. Similarly, Gao et al. [17] noted that AI algorithms struggle to detect minimally displaced or fine cortical fractures. The primary reasons for this include the very fine fracture line on radiographs, low contrast difference with adjacent anatomical structures, and limitations in image resolution [18]. Furthermore, the presence of surgical requirement in 17.39% of FN cases indicates that misdiagnoses can have clinically significant consequences. The high false-negative rates observed particularly in the ankle and foot regions (30.4% and 17.4%, respectively) can be attributed to a confluence of anatomical and radiological challenges specific to these areas. The structural complexity arises from the superimposition of multiple small and irregularly shaped bones, which creates a network of overlapping cortical lines that can obscure subtle, non-displaced fractures. Furthermore, the inherently low soft-tissue contrast in the distal extremities makes it difficult for the AI to distinguish a fine fracture line from a normal trabecular pattern, vascular channel, or artifact. The finding that 100% of the diagnostic disagreements in ankle trauma were false-negatives further highlights that the AI’s current limitation in this region is a systematic failure to detect fractures rather than a tendency to over-diagnose. Across the cohort, false-negative errors outnumbered false-positive errors, suggesting a conservative decision behavior with a tendency toward under-calling fractures; formal calibration analysis was not feasible because the model provided only categorical outputs without probabilistic scores. This difficulty is compounded for fine cortical fractures, which may exhibit only a minimal discontinuity in the cortical line without significant displacement. These factors highlight that the model’s performance is not uniform and is significantly influenced by regional anatomy. Consequently, this finding underscores the critical importance of high image quality and the value of multi-angular evaluations (e.g., additional oblique views) in improving diagnostic accuracy for radiographs of the ankle and foot. Although AP and lateral radiographs from the same patient were provided simultaneously to the multimodal LLM, the model does not perform explicit geometrical multi-view fusion or anatomically aligned cross-projection reasoning as implemented in dedicated multi-view fracture-detection networks. Instead, the projections are processed as paired visual inputs without true 3D-aware spatial correspondence. The lack of such integrated multi-view reasoning may limit the detection of subtle or non-displaced fractures, particularly in anatomically complex regions such as the ankle and foot, and may partly explain the higher false-negative rates observed in these regions. Future studies should investigate architectures or input strategies that enable explicit cross-projection fusion and anatomically registered multi-view analysis. The potential of AI in clinical practice cannot be ignored. Its ability to shorten radiological evaluation time, reduce physician workload, and provide a second opinion during peak hours can offer valuable contributions, especially in high-paced units like the emergency department [19,20]. However, in its current state, the use of AI systems as independent diagnostic tools does not seem appropriate. Our findings indicate that AI is not yet suitable as an independent diagnostic tool but could be integrated as a preliminary assessment or triage system. In such a workflow, the AI could prioritize studies with a high probability of fracture for immediate specialist review and flag uncertain cases for additional scrutiny, thereby potentially reducing time-to-diagnosis for critical cases and helping to manage workload in a busy emergency setting. Nevertheless, the final diagnosis and clinical decision-making must unequivocally remain the responsibility of a specialist physician.

In a safety-oriented ED workflow, the LLM should be implemented strictly as decision support. Cases labeled “Fracture present” could be prioritized for expedited radiologist/orthopedist review. “Uncertain” outputs should trigger mandatory secondary review and/or additional imaging (e.g., additional projections or cross-sectional imaging when clinically indicated). Importantly, “No fracture” outputs should not be used as a stand-alone rule-out; standard clinician interpretation and discharge instructions (including return precautions) should remain unchanged. All outputs should be audit-logged, and clinicians must retain full authority and responsibility for final diagnosis and management decisions.

Because the evaluated multimodal LLM is accessed via a cloud-based API and is not approved as a medical device, its use raises important regulatory and medicolegal considerations. The system does not currently fall under certified diagnostic software categories (e.g., FDA-cleared or CE-marked medical devices), and therefore cannot be deployed for autonomous clinical decision-making. Any future clinical implementation would require formal regulatory approval, compliance with data-protection and cybersecurity standards, institutional governance, and clearly defined liability frameworks. Until such conditions are met, the model should be regarded strictly as an investigational decision-support tool, with full diagnostic and legal responsibility residing with the treating physician.

### 4.1. Technological Development and Clinical Integration

The performance of AI systems largely depends on the quality and diversity of the datasets on which they are trained. Although the model used in our study did not possess an extensive clinical database, it was tested with radiographs obtained from a real clinical environment. The literature suggests that heterogeneous datasets from different populations enhance the generalization capability of AI algorithms [21,22]. During the clinical integration process, it is recommended that these systems be periodically updated and retrained with data specific to the institution’s own patient population. This may enable the reduction in error rates, especially in challenging diagnostic areas like non-displaced fractures.

### 4.2. Ethical and Legal Dimensions

The proliferation of AI-assisted diagnostic systems brings forth debates on ethical and legal responsibility. The fact that some false-negative cases required surgical intervention indicates that misdiagnosis can lead to serious patient safety issues. At this point, it is critically important to position AI solely as a supportive tool, with the final decision always requiring physician approval [23]. Additionally, the use of medical data in AI systems must be carefully managed in terms of data privacy and patient confidentiality. Regulations such as the European Union’s General Data Protection Regulation (GDPR) and similar national frameworks provide binding guidelines for the use of such technologies [24]. Because assessments were performed through a cloud-based API, compliance requires strict governance of data minimization, anonymization, secure transfer, and access control. In this study, AI outputs were generated retrospectively and were not used for real-time clinical decisions; therefore, clinical responsibility remained entirely with the treating clinicians. Any future prospective deployment would require institution-specific legal review, vendor due diligence, and clearly defined accountability pathways.

### 4.3. Clinical Importance of Non-Displaced Fractures

The majority of FN cases in our study consisted of non-displaced fractures. These fractures, due to their minimal radiological signs, can be easily missed by both humans and artificial intelligence. The literature reports that missed non-displaced fractures can lead to prolonged healing due to diagnostic delay, additional surgical requirements, and negatively impacted long-term functional outcomes [25,26]. Therefore, it is necessary to specifically optimize AI detection algorithms for these types of fractures, particularly in high-risk anatomical regions.

### 4.4. The Role of Large Language Models in Image Interpretation

Large language models like LLMs are newly acquiring the capability to process image data directly and currently demonstrate limited performance compared to traditional deep learning-based image analysis systems [27]. However, multimodal AI approaches that jointly assess text, image, and clinical data have the potential to increase diagnostic accuracy. Recent studies have reported that these hybrid systems can achieve accuracy rates comparable to, or in some cases higher than, experienced clinicians [28].

### 4.5. Clinical Integration and Future Perspective

For the successful integration of AI systems into emergency orthopedic practice, multi-center, prospectively designed validation studies are needed. A practical next step is a prospective multi-center reader-study in which emergency physicians and/or radiologists interpret musculoskeletal radiographs under two conditions—standard care (no AI) versus AI-assisted interpretation (LLM used strictly as decision support)—with randomized case order and predefined handling rules for ‘Uncertain’ outputs. Primary endpoints should include sensitivity and false-negative rates for clinically significant fractures (including surgically treated injuries), stratified by anatomical region, while secondary endpoints may include time-to-decision, downstream imaging utilization (repeat radiographs/CT/MRI when clinically indicated), inter-reader agreement, and ED operational metrics. Furthermore, regular performance audits, user training, and clear definition of legal/ethical frameworks are critically important during the clinical integration process [29].

Several strategies may improve the diagnostic performance and generalizability of AI-based fracture detection systems. These include expansion of training datasets through multi-center data collection across different imaging devices and protocols, improvement of label quality via multi-reader adjudication, mitigation of class imbalance with robust data augmentation and standardized preprocessing pipelines, and external validation with prospective study designs. In addition, calibration and threshold optimization, as well as uncertainty-aware and human-in-the-loop deployment strategies, may enhance safety and clinical applicability. Accordingly, any potential clinical use should be considered exploratory and contingent on prospective validation and local governance, rather than interpreted as evidence of near-term clinical readiness.

### 4.6. Limitations

This study has several limitations. Firstly, the research is single-centered and has a retrospective design. This may limit the generalizability of the findings. Additionally, the format, resolution, and acquisition protocols of the examined radiographs are specific to a single center; different imaging parameters in different institutions may affect diagnostic performance. This was a single-center retrospective study, and institutional imaging protocols, patient mix, and documentation practices may differ across settings, limiting external validity. In addition, because the AI assessments were performed retrospectively, we could not evaluate operational outcomes such as changes in clinician behavior, workflow efficiency, or time-to-diagnosis. Prospective multi-center studies are therefore needed to confirm generalizability and to quantify clinical impact within routine emergency department imaging workflows. We did not perform ROC/AUC analyses, which may limit direct comparability with some prior fracture-detection studies that report threshold-based performance curves.

Secondly, the reference standard was based on specialist radiograph interpretation without systematic CT/MRI confirmation. While this mirrors real-world ED practice where initial management decisions rely on radiographs, it is a study limitation. The most subtle fractures (e.g., occult scaphoid or talar fractures) could be missed by both the specialists and the LLM. Therefore, our sensitivity and false-negative rates reflect the LLM’s performance relative to specialist radiograph reading, not to an absolute imaging gold standard.

Thirdly, our study cohort was limited to patients referred from the ED to orthopedics, representing a population with higher pre-test probability for fracture than an unselected ED screening population. This limits the direct generalizability of our predictive values (PPV, NPV) to broader ED settings with lower fracture prevalence. Because several anatomical subgroups had limited sample sizes, their region-wise estimates are associated with wider 95% confidence intervals and should be interpreted as descriptive. Accordingly, a formal post hoc power calculation was not performed; precision was instead assessed using the width of the confidence intervals. Because subgroup and regional analyses were exploratory and not adjusted for multiple comparisons, chance findings cannot be excluded and results should be interpreted as hypothesis-generating.

The evaluated multimodal LLM provided only categorical outputs (“Fracture present”, “No fracture”, “Uncertain”) via its official API, without probability scores, confidence estimates, or exposed logits. Consequently, receiver operating characteristic (ROC) analysis, area under the curve (AUC) calculation, and systematic evaluation of alternative decision thresholds could not be performed, limiting direct comparison with prior task-specific fracture-detection AI studies that report threshold-based performance metrics. To partially address this, we performed a decision-rule sensitivity analysis by reclassifying “Uncertain” outputs as positive, illustrating the sensitivity–specificity trade-off; however, this does not substitute for a full threshold-sweep evaluation. Future studies would benefit from multimodal models that expose continuous output scores or allow adjustable operating points to enable standard ROC/AUC benchmarking.

The AI system used in the study (LLM) operates as a ‘black box,’ and detailed information about its internal decision-making process, training data, and optimization is not accessible. Therefore, the root causes of its performance limitations, particularly in detecting subtle radiological findings like non-displaced fractures, remain inexplicable. This lack of transparency and interpretability represents a significant barrier to clinical trust and adoption, as physicians cannot verify the AI’s reasoning, especially in critical or borderline cases. Future studies should prioritize the development and evaluation of explainable AI (XAI) techniques and models trained on larger, curated multimodal datasets that include detailed anatomical labels to improve not only diagnostic accuracy but also the interpretability of the results.

Radiographic quality was not analyzed as a separate variable in this study because image acquisition was intentionally standardized by design. Specifically, examinations with missing standard projections (anteroposterior and lateral) or insufficient technical quality were excluded prior to analysis, thereby minimizing quality-related variability within the cohort. Nevertheless, future prospective studies could incorporate a predefined radiographic quality scoring system to quantify residual variability in positioning and acquisition parameters and to formally evaluate their association with AI false-negative assessments. Although STARD-AI principles were followed where feasible, full adherence was limited by the retrospective design and the restricted access to proprietary model details.

## 5. Conclusions

In this study, the performance of an AI (LLM)-based diagnostic system was compared against specialist orthopedic physician diagnoses in 1136 patients consulted from the emergency department to orthopedics. The overall agreement rate was 71.13%, and Cohen’s kappa value was 0.634. Regional assessments revealed the highest agreement in knee trauma, while relatively lower agreement rates were detected in shoulder and ankle trauma. The finding that the vast majority (82.61%) of false-negative cases consisted of non-displaced fractures is noteworthy.

The obtained data indicate that AI can provide speed and standardization in emergency orthopedic evaluations but still has significant deficiencies, particularly in detecting non-displaced fractures. The presence of surgical requirements in a portion of the false-negative cases reveals that these deficiencies can directly impact patient safety. Missing non-displaced fractures can lead to delays in the treatment process, additional surgical interventions (e.g., due to a non-displaced fracture transforming into a displaced fracture under load when undetected), and long-term functional losses. Accordingly, our findings should be interpreted as hypothesis-generating evidence on the potential role of a general-purpose multimodal LLM for decision support, rather than as benchmarking against state-of-the-art task-specific fracture-detection models. At this stage, AI systems should not be used as independent diagnostic tools but as auxiliary support systems assisting specialist physician evaluation.

In the future, multimodal AI models trained on broader and more heterogeneous datasets, possessing high-resolution image analysis capabilities, are expected to increase clinical accuracy. Alongside multi-center, prospectively designed validation studies, the development of specialized algorithms to enhance AI performance in different anatomical regions is important. In conclusion, while AI like LLMs shows significant promise as a supportive tool to enhance efficiency in emergency orthopedics, our findings unequivocally demonstrate that human expertise remains the indispensable cornerstone of accurate diagnosis and patient safety, particularly for the subtle fractures that carry significant clinical consequences.

## Figures and Tables

**Table 1:** Overall diagnostic agreement between LLM and orthopedic specialists.

Metric	Value
Number of cases analyzed	1136
Number of fully matched cases	808
Overall agreement rate (%)	71.13
95% Confidence Interval (Wilson)	68.42–73.69
Cohen’s kappa (κ)	0.634

**Table 2:** Diagnostic performance metrics of LLM in detecting orthopedic fractures.

Metric	Value (%)	95% Confidence Interval
Sensitivity	76.9	73.5–80.1
Specificity	66.8	63.2–70.2
Positive Predictive Value (PPV)	69.4	66.0–72.6
Negative Predictive Value (NPV)	74.5	71.2–77.5
Accuracy	71.1	68.4–73.7
F1-Score	0.72	---
Cohen’s κ	0.634	0.60–0.67

**Table 3:** Region-based diagnostic agreement (*n* ≥ 30 cases).

Region	Case Count	Agreement (*n*)	Agreement (%)	95% CI	Kappa	FP
Knee Trauma	96	88	91.67	84.41–95.72	0.710	0
Wrist Trauma	264	208	78.79	73.46–83.29	0.705	40
Hand Trauma	184	128	69.57	62.57–75.76	0.652	16
Shoulder Trauma	104	72	69.23	59.81–77.28	0.532	16
Hip Trauma	48	32	66.67	52.54–78.32	0.5	8
Elbow Trauma	136	88	64.71	56.37–72.23	0.562	16
Ankle Trauma	120	56	46.67	37.98–55.56	0.205	0
Foot Trauma	72	32	44.44	33.54–55.91	0.196	8

**Table 4:** Diagnostic Agreement by Time of Presentation.

Time Period	Case Count	Agreement (*n*)	Agreement (%)	95% CI	Kappa
Daytime (08–19)	592	432	72.97	69.26–76.39	0.639
Night (20–07)	544	376	69.12	65.11–72.85	0.624

**Table 5:** Distribution and characteristics of false-negative (FN) cases.

Metric	Count	Percentage
Total FN	184	16.2%
Non-displaced within FN	152	82.61%
Displaced within FN	32	17.39%
Requiring surgery within FN	32	17.39%
Not requiring surgery within FN	152	82.61%

## Data Availability

The datasets generated and/or analyzed during the current study are not publicly available due to patient confidentiality and institutional data protection policies, but may be made available from the corresponding author (Sadık Emre Erginoğlu) on reasonable request and with permission from the Ankara Sincan Training and Research Hospital Ethics Committee. Access to de-identified data, where permissible, would require institutional approval and a data-sharing agreement consistent with applicable data protection regulations.

## Supplementary Materials

The following supporting information can be downloaded at: https://www.mdpi.com/article/10.3390/diagnostics16030476/s1. Supplementary Table S1. Overall fracture prevalence and confusion matrix for the binary analysis set (excluding ‘Uncertain’ outputs). Supplementary Table S2. Region-wise disagreement profile (false-positive and false-negative counts). Supplementary File S3. STARD-AI checklist.

## Abbreviations

AI	Artificial Intelligence
LLM	Large Language Model
FN	False Negative
PPV	Positive Predictive Value
NPV	Negative Predictive Value
ED	Emergency Department
CI	Confidence Interval
κ	Cohen’s Kappa
