# Supplementary material for: Multimodal Large Language Model for Fracture Detection in Emergency Orthopedic Trauma: A Diagnostic Accuracy Study
Source: Diagnostics (Basel). 2026 Feb 3;16(3):476. doi: 10.3390/diagnostics16030476 (PMC12896714; doi:10.3390/diagnostics16030476)
Supplement: Supplementary file 1 [file diagnostics-16-00476-s001.zip › Supp.3 STARD.pdf]

Item	STARD-AI reporting item (short)	Where addressed in manuscript
1	Identification as a diagnostic accuracy study	Title; Abstract
2	Structured abstract (objectives, design, methods, results, conclusions)	Abstract
3	Scientific and clinical background; intended use	Introduction
4	Study objectives and hypotheses	Introduction; Methods 2.4
5	Study design, setting, and time period	Methods 2.1
6	Eligibility criteria	Methods 2.1
7	Participant selection (consecutive series)	Methods 2.1
8	Participant flow diagram	Figure 1; Methods 2.1
9	Index test definition	Methods 2.2
10	AI model details (type, interface, outputs)	Methods 2.2
11	Image preprocessing and standardization	Methods 2.2
12	Decision thresholds and handling of "Uncertain" outputs	Methods 2.4; Results 3.1
13	Reference standard	Methods 2.3
14	Rationale for reference standard	Methods 2.3
15	Blinding of reference standard to AI results	Methods 2.3
16	Independence of AI assessment from clinical readers	Methods 2.2
17	Handling of indeterminate results	Methods 2.4; Results 3.1
18	Handling of missing or excluded data	Methods 2.1
19	Sample size and rationale	Methods 2.1; Methods 2.4
20	Statistical methods and confidence intervals	Methods 2.4
21	Subgroup and exploratory analyses	Methods 2.4; Limitations 4.6

Item	STARD-AI reporting item (short)	Where addressed in manuscript
22	Participant characteristics	Methods 2.1; Results
23	Disease severity and clinically relevant subgroups	Results 3.2
24	Cross-tabulation of index test vs reference standard	Results Tables; Supplementary Tables
25	Accuracy estimates with precision	Results Tables
26	Adverse events / potential harms	Methods 2.2
27	Study limitations and sources of bias	Limitations 4.6
28	Clinical interpretation and intended role of AI	Discussion; Conclusions
29	Registration and protocol availability	Ethics / End matter
30	Funding, conflicts of interest, data availability	End matter
