# Supplementary material for: Multimodal Large Language Model for Fracture Detection in Emergency Orthopedic Trauma: A Diagnostic Accuracy Study
Source: Diagnostics (Basel). 2026 Feb 3;16(3):476. doi: 10.3390/diagnostics16030476 (PMC12896714; doi:10.3390/diagnostics16030476)
Supplement: Supplementary file 1 [file diagnostics-16-00476-s001.zip › supp.tableS1.pdf]

Supplementary Table S1. Overall fracture prevalence and confusion matrix for the binary analysis set (excluding 'Uncertain' outputs).

Reference Standard \ LLM Output	Fracture Present	No Fracture	**Total**
**Fracture Present**	440 (TP)	192 (FN)	**632**
**No Fracture**	104 (FP)	384 (TN)	**488**
**Total**	**544**	**576**	**1,120**

Overall fracture prevalence (reference standard): 632 / 1120 = 56.4%
