# Supplementary material for: Multimodal Large Language Model for Fracture Detection in Emergency Orthopedic Trauma: A Diagnostic Accuracy Study
Source: Diagnostics (Basel). 2026 Feb 3;16(3):476. doi: 10.3390/diagnostics16030476 (PMC12896714; doi:10.3390/diagnostics16030476)
Supplement: Supplementary file 1 [file diagnostics-16-00476-s001.zip › supp.tableS2.pdf]

Supplementary Table S2. Region-wise disagreement profile (clinically actionable error context).

Region	N	Agreement (n)	FP (n)	**FN (n)***	**Disagreement (FP+FN)**
Knee Trauma	96	88	0	**8**	8
Wrist Trauma	264	208	40	**16**	56
Hand Trauma	184	128	16	**40**	56
Shoulder Trauma	104	72	16	**16**	32
Hip Trauma	48	32	8	**8**	16
Elbow Trauma	136	88	16	**32**	48
Ankle Trauma	120	56	0	**64**	64
Foot Trauma	72	32	8	**32**	40

***FN (n) = N − Agreement (n) − FP (n). This represents the false-negative component of regional disagreement, providing a transparent error profile.*
